# Post-elimination surveillance in formerly onchocerciasis endemic focus in Southern Mexico

**DOI:** 10.1371/journal.pntd.0008008

**Published:** 2020-01-30

**Authors:** Nadia A. Fernández-Santos, Thomas R. Unnasch, Isabel C. Rodríguez-Luna, Francisco Gibert Prado-Velasco, Adebiyi A. Adeniran, Humberto Martínez-Montoya, Mario A. Rodríguez-Pérez

**Affiliations:** 1 Instituto Politécnico Nacional, Centro de Biotecnología Genómica, Reynosa, Tamaulipas, México; 2 Center for Global Health Infectious Diseases Research, University of South Florida, Tampa, Florida, United States of America; 3 Secretaría de Salud del Estado de Chiapas, Tuxtla Gutiérrez, Chiapas, México; 4 Unidad Académica Multidisciplinaria Reynosa Aztlan—Universidad Autónoma de Tamaulipas, Reynosa, Tamaulipas, México; Michigan State University, UNITED STATES

## Abstract

**Background:**

All formerly endemic communities of the Southern Chiapas focus of onchocerciasis in Mexico were treated with ivermectin until parasite transmission was eliminated by 2015. Transmission of onchocerciasis did not resume during a period of three years (2012–2014) following the final distribution of ivermectin in 2011; it was thus concluded that transmission remained undetectable without intervention. WHO thus declared the elimination of transmission of onchocerciasis from Mexico in 2015.

**Methodology/Principal findings:**

From 2016 to the present, post-elimination surveillance (PES) based on examination for suspected onchocercomas was performed in the former Southern Chiapas focus. Each year, over 60% of the total population (range = 85,347–104,106 individuals) of the formerly endemic communities were examined for onchocercomas. Thirty-four individuals were found harboring suspected onchocercomas in the PES surveys conducted from 2016–2019. Of these, one female of 7 years of age who had immigrated from a formerly endemic focus, harbored an infertile (sterile) female in the suspected onchocercoma; all others were negative. Skin biopsy assessments were performed from March through May 2017 in three communities where the female resided. None of the 83 individuals of the three communities examined by skin biopsy were mf positive. Similarly, none of the biopsies from the individuals were found to contain parasite DNA when tested by polymerase chain reaction-enzyme-linked immunosorbent assay (PCR-ELISA).

**Conclusions/Significance:**

These provide support to the conclusion that onchocerciasis has been eliminated from Mexico.

## Author summary

Elimination of transmission of onchocerciasis from Mexico was officially declared in 2015. In the present study, we report post-elimination surveillance (PES) in a formerly onchocerciasis endemic focus in Southern Mexico. From 2016 to the present, PES based on examination for suspected onchocercomas and parasitological studies were performed that together indicate that transmission of *O*. *volvulus* no longer exists in this former focus. This study supports the conclusion that onchocerciasis has been eliminated from Mexico.

## Introduction

Onchocerciasis is transmitted by black flies and has historically been a very serious problem in the developing world, primarily in sub-Saharan Africa and to a lesser extent in 13 foci in 6 countries in Latin America; transmission has now been interrupted or eliminated in 11 of these 13 endemic foci [1–8]. Onchocerciasis has officially been declared to have been eliminated in Guatemala, Colombia, Ecuador and Mexico [2]. The Soconusco (Southern Chiapas)-Huehuetenango focus, located in a region spanning the border between Guatemala and Mexico, was epidemiologically the most important focus of onchocerciasis in the Americas. Mexico was verified as having eliminated onchocerciasis in 2015, followed by Guatemala in 2016 [2, 9, 10]. Transmission was interrupted in 2011 in the formerly endemic Southern Chiapas focus of Mexico and it was subsequently declared eliminated in 2015 following a post treatment surveillance period [9, 11].

In Mexico, post-elimination surveillance (PES) is performed following the Specific Action Program (SAP) entitled "Elimination of Onchocerciasis 2013–2018" issued by the Ministry of Health. This document recommends PES continue after elimination has been verified by WHO, in order to detect possible recrudescence [12–20]. To accomplish this goal, the surveillance of subcutaneous masses suspected of being nodules or onchocercomas has been maintained since 2016. During 2016, the program examined 88,273 individuals in the former Southern Chiapas focus (Table 1). A single nodule containing an infertile female parasite was found in one seven year old female. The female was an inhabitant of the historically hyperendemic community of Nueva Reforma (NR) (Table 2) [11]. The epidemiological follow up of the case revealed that she likely acquired the infection when living in other formerly hyperendemic communities known as Laguna Arenal & Caballo Blanco (LA & CB) in the former Southern Chiapas focus.

**Table 1:** Total population in the formerly Southern Chiapas focus and number of individuals examined for suspected onchocercomas during the post-elimination surveillance (PES).

Year	Total population	No. of individuals examined	Coverage in percent
2016	134,829	88,273	65
2017	137,696	94,463	69
2018	141,413	104,106	74
2019	142,203	85,347	60

**Table 2:** Number of suspected cases of onchocerciasis in the formerly endemic focus of Southern Chiapas, Mexico from 2016–2018.

Year	Municipality	Community	Number of suspected cases of onchocerciasis	Result
2016	Escuintla	Ampliacion Las Malvinas	1	Negative
2016	Escuintla	Barrio El Jilgerillo	2	Negative
2016	Huixtla	Canton El Consuelo	2	Negative
2016	Acacoyagua	Col. Constitucion	2	Negative
2016	Siltepec	Col. Independencia	1	Negative
2016	Acacoyagua	Col. Los Cacaos	1	Negative
2016	Acacoyagua	Col. Nueva Libertad	2	Negative
2016	Acacoyagua	Nueva Reforma	2	1 Negative / 1 Positive (infertile *O*. *volvulus* female)
2017	Montecristo de Guerrero	La Lucha and Col. Toluca	2	ND
2018	Siltepec	Barrio El Joval	1	Negative
2018	Siltepec	Barrio La Lucha	2	Negative
2018	Siltepec	Barrio Llano Grande	4	Negative
2018	Siltepec	Barrio Matasono	11	Negative
2018	Siltepec	Barrio Villaflores	1	Negative
2019			0	
**Overall**			**34**	

ND: Nodulectomy was not performed in the two suspected individuals (a female aged 7 and a male aged 33).

Based upon this finding, a cross-sectional parasitological study based on skin biopsies was performed in NR, LA, and CB in 2017. The study was carried out in coordination with the National Center for Preventive Programs and Disease Control (CENAPRECE) at the Federal level, the General Directorate of Epidemiology at the Federal level, the State Health Institute of Chiapas, the Sanitary Jurisdiction of Tapachula, with the coordination of the Onchocerciasis Program and the National Polytechnic Institute IPN (Center for Genomic Biotechnology). Here, we report the findings of this study.

## Materials and methods

### Study area

**Fig 1:**
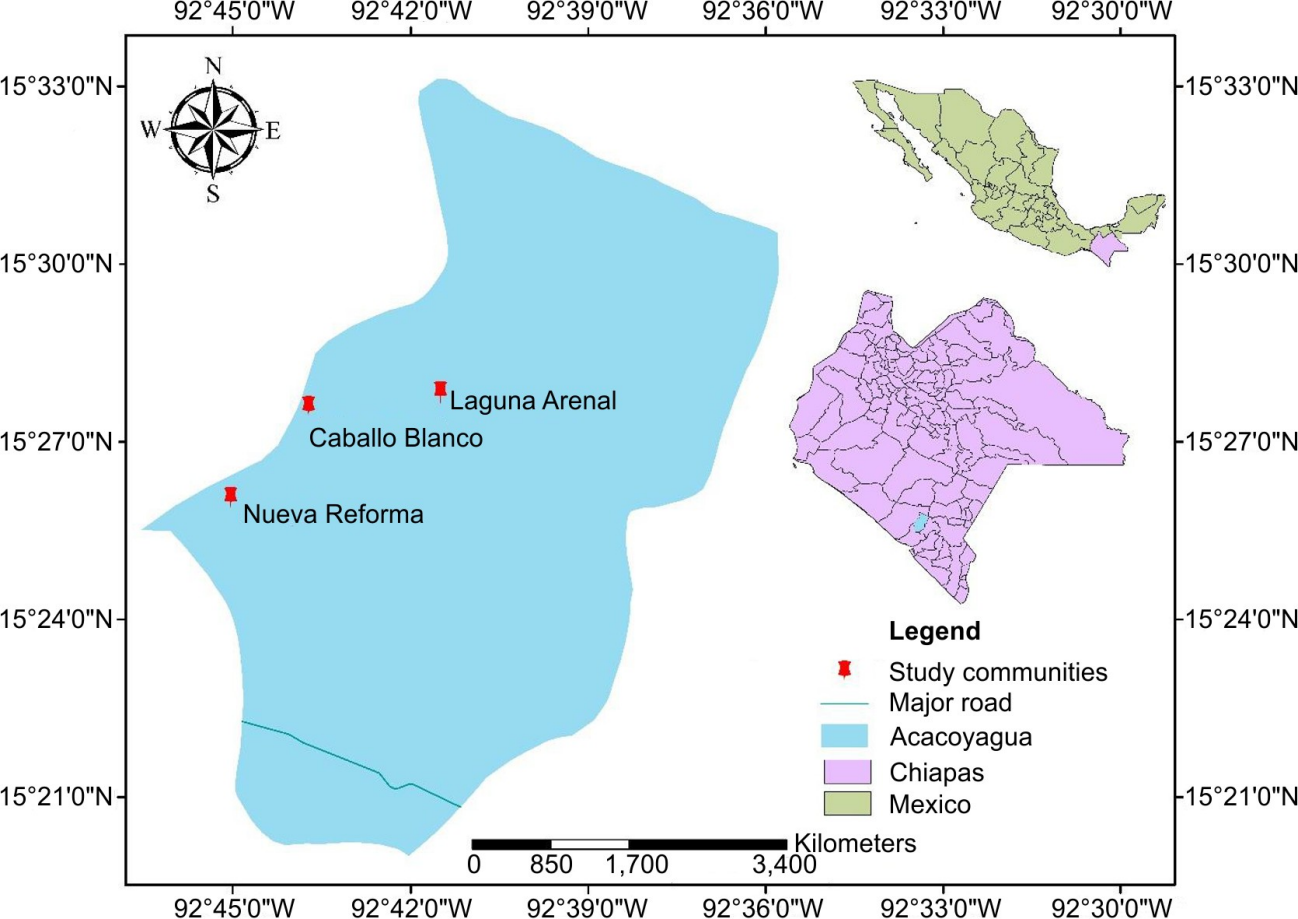
Map showing the study area in the formerly Southern Chiapas focus for onchocerciasis in Mexico. Map of Fig 1 was created using ArcGIS software by ESRI (www.esri.com). ArcGIS and ArcMap are the intellectual property of Esri and are used herein under license. Copyright Esri. All rights reserved. For more information about Esri software, please visit www.esri.com.

**Fig 2:**
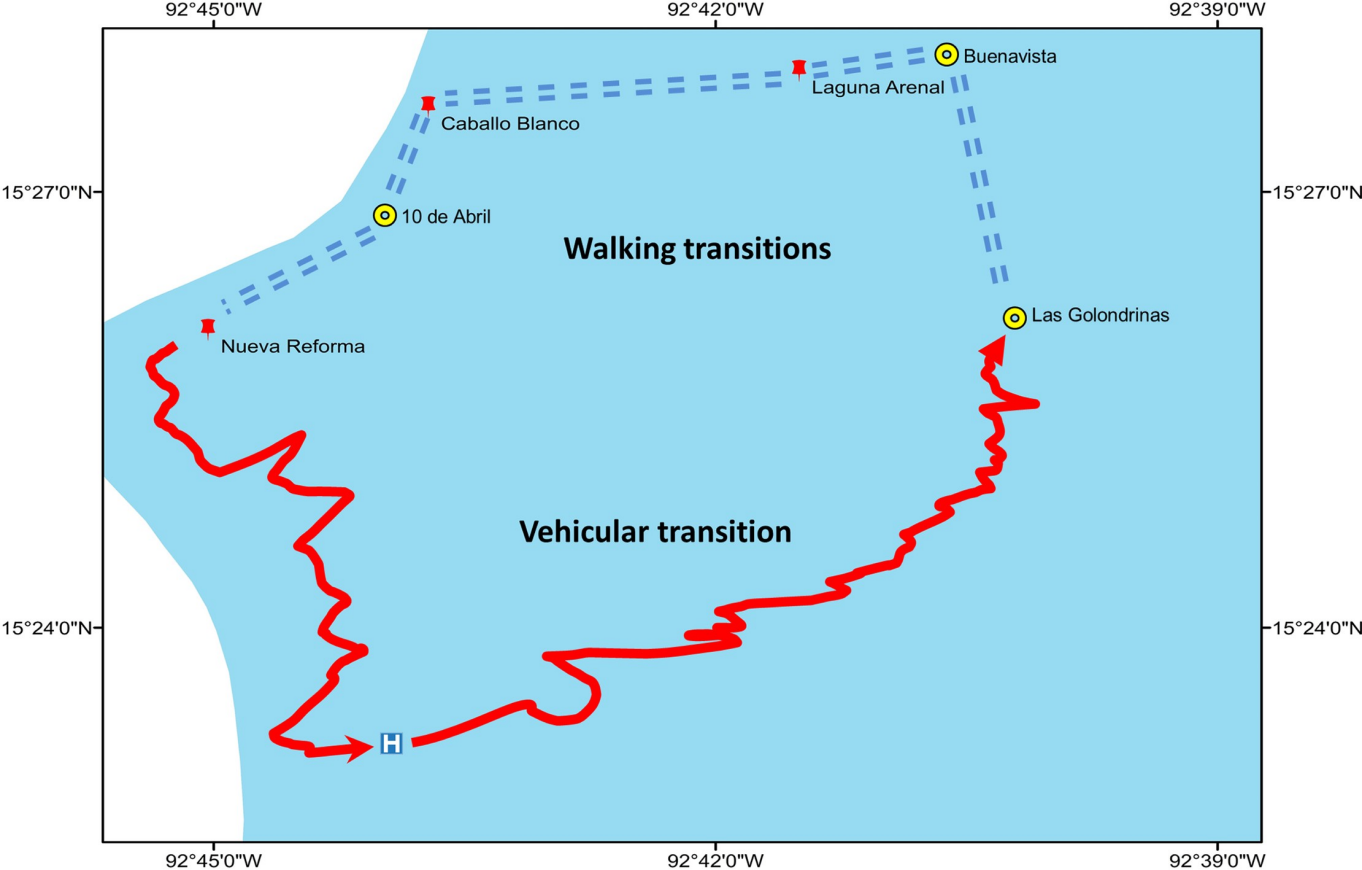
Map showing the walking and vehicular transitions taken for onchocerciasis brigades to reach the three communities under study. Map of Fig 2 was created using ArcGIS software by ESRI (www.esri.com). ArcGIS and ArcMap are the intellectual property of Esri and are used herein under license. Copyright Esri. All rights reserved. For more information about Esri software, please visit www.esri.com.

Three communities of the municipality of Acacoyagua were selected in the former Southern Chiapas focus (Fig 1). These included Nueva Reforma (NR), Laguna Arenal (LA) and Caballo Blanco (CB). NR is located at 92^o^45’02”W, 15^o^26’03”N; altitude 481m (Fig 1). It has 347 inhabitants (192 men and 155 females); 98 were under 10 years old (45 under 5 years old). LA is located at 92°41'30"W, 15°27'50”.N; altitude 120m; it has 161 inhabitants and CB at 92°43’43”W, 15^o^27’35”N; altitude 1295m; it has 36 inhabitants (18 men and 18 females). NR was formerly classified as hyper-endemic for onchocerciasis, and EA and CB are communities which are remote and difficult to access by road (Fig 2). No basic health services are provided in these communities, so residents need to walk several hours to reach the nearest community that provides basic health services (Fig 2).

### Onchocercoma inclusion criteria

The sampling of individuals with suspected onchocercomas was based on individuals at risk; these individuals live in locations previously referred to as endemic. Since 1930, the nodulectomy campaign against onchocerciasis has been ongoing. Removal of nodules was performed on the entire population that lived in the onchocerciasis foci, regardless of age or gender; this was done in a public area in view of the entire community. In the SAP of 2013, the Ministry of Health established the goal of clinically confirming the elimination of onchocerciasis, which implied maintaining surveillance of subcutaneous masses suspected of onchocerciasis in the local populations as well as of migrant coffee workers and residents who went to live elsewhere and have returned to their original community. This surveillance is done annually and has been carried out by the onchocerciasis brigades from 2013 to date. The activity includes educational activities with local health teams and with the communities, to promote the report of the appearance of any subcutaneous mass suspected of being an onchocerciasis nodule; ensuring the review of the masses by personnel trained by the brigades to distinguish “masses” from “suspicious masses”; and to remove the masses that are identified as potential onchocerciasis nodules and submit them to a histopathological examination for confirmation.

### Index case description and inclusion criteria

The positive person harboring the suspected onchocercoma was a female seven years old resident of NR. The nodule was noted by her mother in 2015, and the onchocerciasis brigade had its first contact with the patient at the beginning of 2016. The onchocerciasis brigade identified the female as an immigrant since she had only lived for one year in NR. She was originally from the communities of EA and CB. Although the family had seven daughters, only one (the fifth daughter) had the suspected nodule. It was determined that the community where the female is currently residing should be considered as community in which all children under 8 years of age would be studied since they have never received treatment with ivermectin. The last treatment with ivermectin was given during 2011.

### Parasitological study

Four skin snips or biopsies were taken by the onchocerciasis brigades from both left and right shoulders and left and the right supra-scapular region in 36, 24, and 23 patients of NR, LA and CB, respectively (Table 3) using a corneoscleral biopsy instrument. Samples were collected using convenience sample methods and included as many children and participating adult adults as possible during the days of the parasitologic surveys. The index case, a girl living in NRA but that had been living in LA for 2 years, a formerly hyperendemic community that had treatment with ivermectin for over 15 years; local health authorities agreed that the girl, the closest family relatives, and most children that have not been treated with ivermectin would be biopsied. The girl also came from a more remote community CB that also was hyperendemic; thus this community was also included in the parasitological surveys. No children or parents refused to be examined but absent persons were not included in the survey. CB is a very remote community and the skin snip samples had to be read in less than 24 hours, so extending the survey past 24 hours was not possible.

**Table 3:** Parasite prevalence (skin mf by microscopy and O-150 PCR-ELISA) in three communities of the formerly Southern Chiapas focus, Mexico.

Community	No. of individuals examined / total population	% parasite point prevalence
Nueva Reforma	36 / 347	0
Laguna Arenal	24 / 161	0
Caballo Blanco	23 / 36	0
**Overall**	**83 / 544**	**0**

The female with the nodule and her relatives were included in the 36 patients from NR. The skin biopsies were then weighed and incubated in 0.2 ml of buffer (normal saline or culture medium) in a microtiter plate. Mf were counted by microscopy at 4, 12 and 24 h after the biopsies were taken. Putative onchocercomas were examined by qualified personnel from the Public Health Laboratory of Chiapas.

### Skin snip PCR to detect parasite DNA

As all snips were microscopically negative, the snips were tested using the O-15O PCR, which is an extremely sensitive and specific test for residual parasite DNA, and does not require the presence of viable or intact parasites to give a positive result [21]. The four skin snips from each individual were pooled and homogenized in 100μl of 10mM Tris-HCl, 1 mM EDTA (pH 8.0), and proteinase K added to a final concentration of 2mg/ml. The homogenate was incubated at 56°C for 2 hours and dithiothreitol added to a final concentration of 20 mM. The samples were boiled to 95°C for 30 minutes to disrupt the parasite cuticle. Boiling was followed by a series of three freeze-thaw steps to release the parasite DNA.

The homogenates were subjected to centrifugation at 13,100 x g for five minutes and the supernatant placed into a new tube. The solutions were brought to a final concentration of 100 mM Tris-HCl (pH 7.5), 100 mM NaCl. A total of 5 μl of a 0.5 mM solution of OVS2-biotin capture probe (5´B-AATCTCAAAAAACGGGTACATA-3´, where B = biotin) was added to each sample. The solutions were then heated to 95°C for three minutes and allowed to cool slowly to room temperature. While the probe was annealing to the DNA in the solution, 10 μl of Dynabeads M-280 streptavidin per sample (Invitrogen, 112-05D) were added to wells of a 96 well plate, with each well containing a maximum of 50 μl of bead solution. The plate was placed on a magnetic capture unit (Magnetic 96-Well Separator, ThermoFisher Scientific, A14179) and the beads collected for two minutes. The beads were then washed five times with 200 μl binding buffer (100 mM Tris-HCl (pH 7.5), 100 mM NaCl) per wash, allowing two minutes between washes for the magnet to re-capture the beads. The beads were then resuspended in the original volume of binding buffer and 10 μl of the bead solution was added to each sample. The samples were incubated on a shaker or roller overnight at room temperature to permit the oligonucleotide-DNA hybrids to bind to the beads.

The samples were placed in the magnetic separator for two minutes to capture the beads and the supernatant discarded. The beads were resuspended in 150 μl of binding buffer by pipetting, and the beads captured by placing the plate in the magnetic separator for two minutes. The wash step was repeated five times, allowing two minutes between washes for the magnet to re-capture the beads. The beads were then resuspended in 20 μl of PCR water, heated to 80°C for two minutes and cooled rapidly on ice for five minutes. The beads were removed by placing the plate in the magnetic capture unit, and the supernatant containing the purified DNA was transferred to a new plate for storage. A total of 5 μl of these DNA solutions were used as template DNA in each PCR reaction. The PCR reaction, PCR conditions, and detection of PCR products by ELISA was essentially the same as that used for the detection of O-150 PCR reactions using black fly DNA as a template [22–24].

### O-150 PCR analysis

A total of 5 μl of the purified genomic DNA was used as a template for the PCR amplifications carried out in a total volume of 50 μl containing a final concentration of 0.5 μM O-150 primer (5′-GATTYTTCCGRCGAANARCGC-3′) and 0.5 μM biotinylated O-150 primer (5′B-GCNRTRTAAATNTGNAAATTC- 3′, where B = biotin; N = A, G, C, or T; Y = C or T; and R = A or G). Reaction mixtures also contained 50 mM Tris-HCl (pH 9.0), 15 mM (NH_4_)_2_SO4, 1.5 mM MgCl_2_, 0.2 mM each of dATP, dCTP, dGTP and dTTP, and 0.05 U of Taq polymerase (Promega). Cycling conditions consisted of five cycles of one minute at 94°C, two minutes at 37°C, and 30 seconds at 72°C, followed by 35 cycles of 30 seconds each at 94°C, 37°C, and 72°C. The reaction was completed by incubating at 72°C for six minutes [22, 25–27]. Amplification products were detected by PCR enzyme-linked immunosorbent assay (ELISA), essentially as previously described [22, 23, 28–30]. Briefly, 5 μl of each PCR reaction was bound to a streptavidin-coated ELISA plate, and the DNA strands denatured by treatment with alkali. The bound PCR fragments were then hybridized to a fluorescein-labeled *O*. *volvulus*-specific oligonucleotide probe (OVS2-FL probe: 5′-AATCTCAAAAAACGGGTACATA-FL3′ where FL = flourescein), and the bound probe detected with an alkaline phosphatase-labeled anti-fluorescein antibody (Anti-Fluorescein-AP Fab fragments; Roche Diagnostics, 11426338910). Bound antibody was detected using the ELISA Amplification System Kit (Invitrogen, USA, 19589–019) following the manufacturer's instructions. Color development was stopped by the addition of 25 μl 0.3M H_2_SO_4_ solution, and the plates read in an ELISA plate reader set at 495 nm. Samples were scored positive if their optical density exceeded either the mean plus three standard deviations of ten negative control wells run in parallel or 0.1, whichever was greater [27].

### Ethics statement

Community meetings were held in all selected villages within the former focus to explain the procedures, and the right of each individual to decide whether or not to participate was explained. The individuals were also informed that they would be provided with the results of the tests upon request. Before each examination, each adult who had voluntarily come to the examination point and agreed to participate were provided with a capsule summary of the project and process and oral consent was obtained. Parents or guardians provided oral consent on behalf of all child participants. The Ethical Committee of the Health Secretariat of México approved the use of oral consent, given that the studies were conducted as part of the national onchocerciasis surveillance program and were therefore part of a routine public health monitoring program conducted by the Mexican government.

## Results

From 2016–2019, PES was performed in the former Southern Chiapas focus. Each year, over 85,000 residents (range = 85,347–104,106 individuals) of the formerly endemic communities were examined for onchocercomas (Table 1) by the brigades.

In the first four-years of PES, 34 individuals were found with suspected onchocercomas (Table 2). The majority of onchocercomas that were not due to onchocerciasis were mostly fat mass (sebaceous). As of 2004, all onchocercomas removed were initially sent to Germany for histopathological analysis. Beginning in 2009, the onchocercomas were sent to a local laboratory and analyzed by personnel trained for this purpose. During the staff training, onchocercomas were sent to the Centers for Disease Control and Prevention in Atlanta for quality control, and the local identifications were found to be 100% reliable. The identification technique used was histopathological analysis under a microscope.

**Fig 3:**
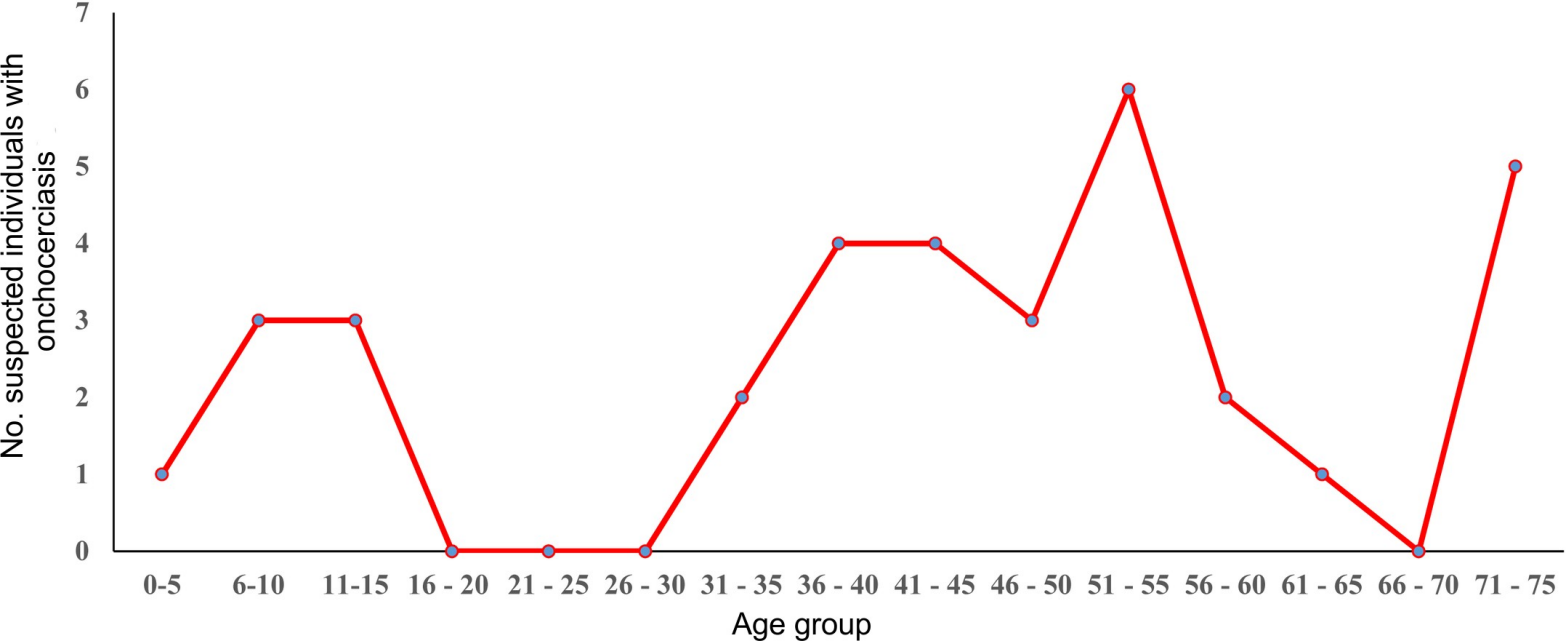
Suspected cases of onchocerciasis by age group in the formerly endemic focus of Southern Chiapas, Mexico, from 2016–2019.

The age groups with the highest number of suspected onchocercomas were 51–55 and 71–75 years old (six and five putative onchocercomas, respectively; Fig 3). Only one individual was found to harbor a verified onchocercoma (a female of seven years of age) by qualified personnel from the Public Health Laboratory of Chiapas. This onchocercoma contained a single infertile female parasite. As discussed above, this finding prompted a more thorough examination of a sample of individuals (n = 83) residing in the infected female’s current and former resident communities of NR and LA and CB. None of the 83 residents examined by skin biopsy were found to have mf or evidence of parasite DNA in these three communities (Table 3). In the most remote community (CB; Fig 2), 23 individuals of total population of 36 was examined by skin biopsy representing a coverage of 64%.

## Discussion

The surveys reported here represent the first reports of post elimination surveillance activities undertaken in the countries in Latin America that have successfully completed a post treatment surveillance period and have been verified by WHO as having eliminated onchocerciasis. In the survey conducted in the formerly hyper endemic communities of Southern Chiapas in Mexico, no evidence for patent (i.e. microfilaria positive) infections were found. However, one individual was found to be infected with a single non-fertile female adult parasite. This was not unexpected, as modeling studies conducted by Davies (1993) suggested that at the end of the treatment regime with ivermectin, there will be a period of 3–5 years during which a limited number of members of a community will continue to be infected with *O*. *volvulus*, but at such a low level as to not result in recrudescence [31]. These members are the residual 2–3% of the population who were not eligible for treatment during the treatment program plus chronic refusers and absentees and the occasional individual who did not respond to treatment with ivermectin. Since these individuals did not receive a proper regime of ivermectin, they can remain infected for a period of up to 10 years (the average estimated lifespan of the adult worms [32]) from the time that transmission was suppressed. The one individual found to be infected fell into this group, as she would have been one year old and ineligible for treatment in 2011, when the last treatments were given in these communities. Thus, individuals who were ineligible to receive ivermectin by the time it was stopped in 2012 should thus be closely monitored.

Although such infected individuals represent a small segment of the overall population, it will be important to identify and monitor them and to attempt to convince them to undergo treatment if possible. While their number and intensity of infection may not pose a risk of igniting recrudescence, the disease cannot be claimed to be permanently eliminated while they are in the community. Thus, the Ministry of Health in Mexico has implemented a PES system to keep track of these potentially infected people and also to record the arrival of any incomers who might be infected (*e*.*g*. old residents returning from cities who were infected on departure and who have escaped regular treatment) in each treated community. These monitoring activities have continued during the post treatment era. Together, the results suggest that the situation found in Southern Chiapas is well within the predictions of the models for what will occur when onchocerciasis has been interrupted, supporting the conclusion that onchocerciasis was eliminated from this focus in 2011.

We could not use standard statistical methods to calculate the 95% exact upper limit confidence intervals for point prevalence of skin mf in the communities as well as those for point prevalence of PCR-ELISA, because we examined a large proportion of the population in each case. Thus, as we sampled as many people as possible, conventional statistical methods do not apply in these cases. A limitation of the present study was that it relied initially on palpation of onchocercomas, which is likely to be insensitive, in an elimination setting such as the formerly endemic focus in Chiapas. Other approaches to PES which have been promoted by the WHO such as black fly surveillance, Ov16 serology, should also be considered for PES in Mexico. As the vector *S*. *ochraceum* s.l. is still biting humans in the formerly endemic foci in Mexico, there should be plans to corroborate these findings with one of these other approaches.

Post-elimination surveillance activity should be undertaken by all countries that have managed to demonstrate the elimination of onchocerciasis. It is vital to ensure early detection of a probable recrudescence of the disease. In Southern Chiapas, this is particularly important as a large number of migrants are found in this area. These come not only from the countries of Central America and Latin America but also from the African continent. It is therefore possible that *O*. *volvulus* infected individuals may be present in this migrant population, presenting a risk for re-introduction and recrudescence in these communities warrant close monitoring of these individuals. Thus, it is important to maintain a permanent PES to guard against this possibility.
